# Nano-Based Theranostic Platforms for Breast Cancer: A Review of Latest Advancements

**DOI:** 10.3390/bioengineering9070320

**Published:** 2022-07-15

**Authors:** Rabia Arshad, Maria Hassan Kiani, Abbas Rahdar, Saman Sargazi, Mahmood Barani, Shirin Shojaei, Muhammad Bilal, Deepak Kumar, Sadanand Pandey

**Affiliations:** 1 Faculty of Pharmacy, University of Lahore, Lahore 54000, Pakistan; rabia.arshad@bs.qau.edu.pk; 2 Department of Pharmacy, Iqra University, Islamabad 44000, Pakistan; maria.kiani@iqraisb.edu.pk; 3 Department of Physics, University of Zabol, Zabol 98613-35856, Iran; 4 Cellular and Molecular Research Center, Research Institute of Cellular and Molecular Sciences in Infectious Diseases, Zahedan University of Medical Sciences, Zahedan 98167-43463, Iran; sgz.biomed@gmail.com; 5 Medical Mycology and Bacteriology Research Center, Kerman University of Medical Sciences, Kerman 76169-13555, Iran; mahmoodbarani7@gmail.com; 6 Imam Ali Hospital, Kermanshah University of Medical Sciences, Kermanshah 67158-47141, Iran; shojaeishirin891@gmail.com; 7 School of Life Science and Food Engineering, Huaiyin Institute of Technology, Huaian 223003, China; bilaluaf@hotmail.com; 8 Department of Pharmaceutical Chemistry, School of Pharmaceutical Sciences, Shoolini University, Solan 173229, India; guptadeepak002@gmail.com; 9 Department of Chemistry, College of Natural Science, Yeungnam University, 280 Daehak-Ro, Gyeongsan 38541, Korea

**Keywords:** breast cancer, cancer imaging, theranostics, nanoparticles, nanotechnology

## Abstract

Breast cancer (BC) is a highly metastatic multifactorial disease with various histological and molecular subtypes. Due to recent advancements, the mortality rate in BC has improved over the past five decades. Detection and treatment of many cancers are now possible due to the application of nanomedicine in clinical practice. Nanomedicine products such as Doxil^®^ and Abraxane^®^ have already been extensively used for BC adjuvant therapy with favorable clinical outcomes. However, these products were designed initially for generic anticancer purposes and not specifically for BC treatment. With a better understanding of the molecular biology of BC, several novel and promising nanotherapeutic strategies and devices have been developed in recent years. In this context, multi-functionalized nanostructures are becoming potential carriers for enhanced chemotherapy in BC patients. To design these nanostructures, a wide range of materials, such as proteins, lipids, polymers, and hybrid materials, can be used and tailored for specific purposes against BC. Selective targeting of BC cells results in the activation of programmed cell death in BC cells and can be considered a promising strategy for managing triple-negative BC. Currently, conventional BC screening methods such as mammography, digital breast tomosynthesis (DBT), ultrasonography, and magnetic resonance imaging (MRI) are either costly or expose the user to hazardous radiation that could harm them. Therefore, there is a need for such analytical techniques for detecting BC that are highly selective and sensitive, have a very low detection limit, are durable, biocompatible, and reproducible. In detecting BC biomarkers, nanostructures are used alone or in conjunction with numerous molecules. This review intends to highlight the recent advances in nanomedicine in BC treatment and diagnosis, emphasizing the targeting of BC cells that overexpress receptors of epidermal growth factors. Researchers may gain insight from these strategies to design and develop more tailored nanomedicine for BC to achieve further improvements in cancer specificity, antitumorigenic effects, anti-metastasis effects, and drug resistance reversal effects.

## 1. Introduction

Breast cancer (BC) is one of the most prevalent cancers in women [1]. This type of malignancy often starts from ductal hyper-proliferation with its expansion into benign tumors or metastatic carcinomas resulting from exposure to carcinogenic agents [2]. This disease exhibits not only a great deal of heterogeneity but also a great deal of variation in its occurrence, treatment response, progression, and even location of metastasis. It is common for metastatic BC to impact several organs, including the brain, lung, and possibly bones [3,4]. BC is the second leading cause of death among women worldwide according to the World Health Organization (WHO) [2,5,6,7]. As indicated by the WHO, in 2020, 2.3 million women were diagnosed with BC, and 685,000 women died from the disease worldwide [8,9].

BC is classified into three main tumor subtypes rooted in enhancement in expression of either progesterone receptor (PR) or estrogen receptor (ER), and/or epidermal growth factor receptor 2 (ERBB2) gene mutation [10,11,12,13,14]. Each type of cancer has a unique risk factor and treatment available [15,16]. For optimal therapy, determining the tumor subtype, cancer stage, and patient-favored treatment is a necessary step that must be taken [10]. Applying systemic treatment for nonmetastatic BC patients depends on the results of the hormone receptor and ERBB2 examinations. According to the available evidence, hormone receptor-positive endocrine therapy and chemotherapy are standard BC treatments [17,18].

The prognosis for women who have developed metastatic triple-negative BC is poor. The use of antibody–drug conjugates (ADCs) has been examined as a viable therapy option, especially in extensively pretreated illnesses. In metastatic triple-negative BC, sacituzumab govitecan is a new antibody–drug conjugate (ADC) that has demonstrated promising results. The FDA has designated it as a breakthrough therapy for treating individuals with previously treated metastatic triple-negative BC. For sacituzumab govitecan to operate, an antibody targeting the most common kind of BC-specific antigen, a human trophoblast cell surface antigen 2 (Trop-2), must be combined with the enzyme inhibitor SN-38 (topoisomerase I inhibitor) through a unique hydrolyzable linker [19,20,21]. Sacituzumab govitecan has a well-defined and manageable toxicity profile, and rapid recognition and appropriate early and proactive management will allow clinicians to optimize sacituzumab govitecan treatment for patients [22]. According to the bystander effect for cancer therapy, the high antibody-to-payload ratio of sacituzumab govitecan, and the very toxic characteristics of the drug’s active ingredient, SN38, make it a desirable candidate [23].

One of the most important advances in treating metastatic BC has been the introduction of anti-HER2 drugs, which have considerably improved survival results. Notwithstanding their effectiveness, it is still necessary to use chemotherapy in conjunction with anti-HER2 monoclonal antibodies. When an antigen-specific antibody is coupled with a robust, cytotoxic payload, the outcome is an increased therapeutic index known as antibody–drug conjugates (ADCs). Creating antibody–drug conjugates, which consist of a cytotoxic agent and a monoclonal antibody carrier, provides a significant alternative to the traditional approaches used in chemotherapy. An anti-human epidermal growth factor receptor 2 (HER-2) antibody, a cleavable tetrapeptide-based linker, and a cytotoxic topoisomerase I inhibitor are the three components that make up the antibody–drug combination known as trastuzumab deruxtecan. Trastuzumab deruxtecan was found to have a promising efficacy in patients with HER2-negative or low-expressing disease, who have few treatment options due to the absence of the HER2 gene. This finding led to favorable outcomes in patients with HER2-positive BC who had undergone extensive prior treatment. The US Food and Drug Administration has awarded trastuzumab deruxtecan rapid approval in advanced or unresectable HER2-positive BC that has been treated with at least two HER2-targeting therapy lines, based on results from recent clinical studies. It is feasible that newer HER2-targeted ADCs might offer a wide range of novel therapeutic uses outside typical HER2-positive BC because of their enhanced pharmacological features, such as the ability to target cells with low expression of the HER2/ERBB2 mutation [24,25]. Furthermore, as a preventive measure, small molecules or antibodies that target the human epidermal growth factor receptor-2 (HER2) along with chemotherapy are effective for patients with ERBB2-positive tumors and, consequently, patients with triple-negative BC (TNBC) require only chemotherapy [10].

There are two options for the surgical phase with similar survival rates: a lumpectomy with radiation if the tumor can be excised completely with good cosmetic results, or a mastectomy [6,10]. There are several challenges and decisions that BC patients face due to treatment-related side effects [26,27,28]. Currently, no concrete evidence shows whether breast-conserving therapy or mastectomy leads to better outcomes [29]. An estimated one-third of patients avoid undergoing formal treatment after primary phases according to multiple observational reports [28,30]. It is crucial to consider the issue of chemotherapy-induced undesired effects on other normal organs, ranging from cardiotoxicity caused by doxorubicin (DOX) administration to peripheral neuropathy and even neurotoxicity, which are the most debilitating long-term side effects in BC survivors [31,32,33]. A major side effect of chemotherapy treatment is neurotoxicity and its associated cognitive manifestations. Clinical studies have focused most extensively on the effect of chemotherapy on cognition in BC patients [34,35]. Radiation therapy has a significant impact on the treatment of BC. Research suggests more than 80% of patients with BC receive it alone or in combination with chemotherapy [36]. Most patients tolerate radiotherapy, but some suffer from radiation-related complications, such as late pulmonary and cardiac side effects [37,38,39].

For health care professionals, screening and diagnosis methods are crucial to supply individualized therapies that will improve outcomes and survival [40,41,42]. Conventional tools for cancer diagnosis, including tissue sampling, mammography, contrast-enhanced mammography, ultrasound, MRI, positron emission tomography, and computerized tomographic examination, are still the most employed methods. However, these expensive techniques involve high radiation exposure [43]. Tissue sampling or breast biopsy is commonly performed to discriminate between benign and tumorous tissues. The method is, however, expensive and calls for the expertise of medical professionals [44]. Mammography and ultrasound are also frequently adopted procedures for BC diagnosis; however, at the expense of high radiation exposure, lesions less than 5 mm stay undetected in mammograms. Unfortunately, the shortcoming associated with mammography is related to false-negative diagnoses and inordinate additional checkups. False-positive diagnosis leads to an unnecessary biopsy, emotional burden, and radiation exposure. Low sensitivity for dense breasts is another negative aspect of mammography [45,46]. Magnetic resonance imaging (MRI) can also be used to detect small lesions if the scan is carried out with sufficient contrast; however, it is expensive and less precise [47]. Positron emission tomography is considered a handy tool for monitoring tumor response to therapy [48]. Another technique, known as microwave imaging, has also been investigated to be helpful in the diagnosis of cancer in experimental settings. In addition, the ablation of benign or malignant BC using image-guided focused ultrasound (FUS) is a non-invasive technique that has been used against BC. Image guidance can be accomplished during the ablation process using real-time ultrasound (US) [49]. Although breast ultrasonography is an auxiliary examination, it has numerous problems such as high dependence on test equipment and testers, false positives leading to extra examinations and biopsy, and clarified lesions impeding early BC diagnosis [50,51]. A generous amount of ongoing research in the field of cancer has offered numerous alternatives for cancer diagnosis [48].

As conventional cancer diagnostics and therapeutic tools become less effective due to increased systemic toxicity, nanotechnology is currently utilized to improve diagnosis and mitigate disease severity [52,53,54]. Acquiring chemotherapeutic resistance in BC has also been one of the most significant challenges researchers face when developing effective chemotherapy [55]. Nanomedicine has offered a groundbreaking and potentially beneficial alternative technology that demonstrates numerous advantages over conventional cancer therapies and paves the way for new opportunities for early detection and improved therapy of BC [56]. Over the past several years, research in the field of cancer nanotechnology has brought about a paradigm shift as a result of fabricating novel lipid nanoparticles (LNPs), smart polymers, lipids, inorganic materials, liposomes, nanotubes, polymer lipid hybrid systems, and subsequent engineering of their surface with targeting ligands [55,57]. In addition, nanotechnology has further advanced and brought to light new theranostic strategies, which simultaneously combine imaging and treatment. BC nanomedicines can deliver chemotherapy drugs while also providing lower systemic toxicity. Still, it is necessary to consider the complexity and dynamics of cancer to bridge the translational bench-to-bedside gap [58]. Although there are different approaches for sensing inside living cells, obtaining robust and accurate results is not a trivial matter, and currently, most intracellular sensing approaches are based on the use of fluorescence microscopy [59]. Nanosensors will permit the early diagnosis of predictive biomarkers of BC and enhance the development of accurate and individualized treatments for BC patients [60]. For example, in creating and manufacturing biosensors and sensors for cancer diagnosis, magnetic nanoparticles (MNPs) have garnered a lot of attention as they can be incorporated into the transducers and/or dispersed throughout the specimen. These MNPs could then be captivated by the active detection surface of the nanosensor by magnetic fields that are external to the device [61,62].

Nanotechnology-based immunotherapy agents have been employed for multiple cancer types to shrink the malignant cancerous cells and preserve the benign cells at the target spot [63,64,65,66,67]. To overcome the insufficient specificity of conventional BC treatments, passive targeting and active targeting using nanomaterials to boost tumor drug levels and reduce noncancer drug levels are being explored as potential alternatives. It has been established that undesirable pharmacokinetics, including quick clearance from the body and short half-life, can be overcome via functionalizing (i.e., PEGylation) of NPs. Dose-limiting toxicity of the chemotherapeutic drugs can be resolved via developing nanocarriers with controlled drug release at tumor sites. While increased drug efflux via cell membrane transports transport can cause drug resistance, some NPs acting as passive/active targeting therapeutic agents can inhibit drug efflux mechanisms, and, therefore, enhance endocytosis of the cargo. More importantly, drug resistance in the tumor microenvironment (TME) due to lower pH or hypoxia can be addressed via the development of stimulus-responsive nanoformulations (i.e., pH-responsive nanosystems) [68].

Multipurpose nanotechnology-based plans with diagnostic imaging and targeted treatment roles have been among the most debated topics in the nanomedical research field [69,70,71,72,73]. Lately, much effort has been put into developing novel nano-based systems for theranostic purposes against BC. To this end, Yubei and coworkers focused on finding a nano-compound with high biocompatibility and improved remedial outcomes for coalescent diagnosis, therapy, and clinical modification. The developed NPs were loaded with Iron (П) phthalocyanine and the aptamer AS1411 as a molecular probe, and the assays demonstrated outstanding potential for BC inhibition [74]. In another experiment, Cabral and coworkers investigated the in vivo impacts of photodynamic treatment of BC using nanoemulsions loaded with both a photosensitizer and a chemotherapeutic agent (DOX). They observed that the combined regimen in the presence of laser radiation markedly inhibited the growth of 4T1-stimulated BC in mice. These findings demonstrate the effectiveness of this therapy, which could potentially replace the presently used anti-cancer techniques [75]. In another experiment, Kim and coworkers proposed aptamer-conjugated lipid nanovehicles entrapping quantum dots (QDs) and siRNA for TNBC management. Their findings revealed that anti-EGFR aptamer-conducted lipid transporters could be a promising nanocarrier device for RNA intervention and fluorescence imaging of these highly invasive BC cells [46,76]. In 2021, Taherian and coworkers characterized chitosan-coated magentic NPs and examined their efficiency as black pomegranate peel extract (PPE) transporters [77]. Their results indicated that the drug-loaded NPs can eradicated cancerous cells significantly compared to that of free drug alone. According to the findings by Khodashenas and coworkers in 2021, the entrapping efficiency of gold nanoparticles (AuNPs) in the corresponding biopolymeric system was improved compared to that of AuNPs alone, and a maximum controlled release of MTX was obtained. Moreover, the prepared formulation exerted significant cytotoxic activity in MCF-7 BC cells, while free MTX exhibited much lower cytotoxicity [78]. More recently, Lentiza and collaborators studied the features of silver telluride Ag_2_Te NPs and their utilization in the biomedical situation demanding the maximum NP dosage (i.e., X-ray imaging). This study’s evidence highlighted the impending utilization of Ag_2_Te NPs in the biomedical arena and as X-ray contrast agents for BC detection [79].

Limitations in conventional diagnostic approaches have resulted in the emergence of novel means, such as nanotechnology, which is expected to positively impact BC patients’ survival and quality of life. There is a level of dependency on estrogen for growth in around 70–75% of BCs, which is shown by the expression of the estrogen receptor (ER) [80]. In fact, BCs overexpressing HER2-receptors (HER2+) account for around 15% of all BC cases [81]. The second most common subtype (approximately 12%) is aggressive triple-negative breast cancer (TNBC) [82]. Interestingly, it has been reported that anabolic and androgenic endocrine treatments (e.g., tamoxifen or aromatine inhibitors) and bilateral oophorectomy may be used to limit estrogen synthesis or impede the activation of estrogen receptors in BC cells that are positive for estrogen receptors (the ER+ subtype) [82]. We believe nanotechnology will provide a novel promising avenue for improving BC detection and treatment by utilizing targeted functional nanostructure and nanosensors. In this review, we hope to shed light on the recent advancements in nanomedicine in BC theranostic emphasizing on targeting BC cells that overxpress receptors of epidermal growth factors.

## 2. Nanostructures for BC Diagnosis

As discussed earlier, BC is the most widely encountered malignancy with a high mortality rate [83]. The usual 5-year endurance of women with BC is closely correlated with the tumor phase (98% of 5-year survival at stages 0–1 compared to 85%, 60%, and 20% for stages 2,3, and 4, respectively) [43]. Early detection of BC can significantly reduce the mortality rate associated with the disease, thus improving management and treatment [83]. Conventional tools for cancer diagnosis, including tissue sampling, mammography, contrast-enhanced mammography, ultrasound, MRI, positron emission tomography, and computerized tomographic examination, are still the most employed methods. However, these expensive techniques involve high radiation exposure [43]. Tissue sampling or breast biopsy is commonly performed to discriminate between benign and tumorous tissues. The method is, however, expensive and calls for the expertise of medical professionals [44]. Mammography and ultrasound are also frequently adopted procedures for BC diagnosis; however, at the expense of high radiation exposure, lesions of less than 5 mm stay undetected in mammograms. An MRI can be used to detect small lesions if the scan is carried out with sufficient contrast; however, it is expensive and less precise [47]. Positron emission tomography is considered a handy tool for monitoring tumor response to therapy [48]. Another technique, known as microwave imaging, has also been demonstrated to be helpful in the diagnosis of cancer in experimental settings. A generous amount of ongoing research in the field of cancer has offered numerous alternatives for cancer diagnosis [48].

**Figure 1:**
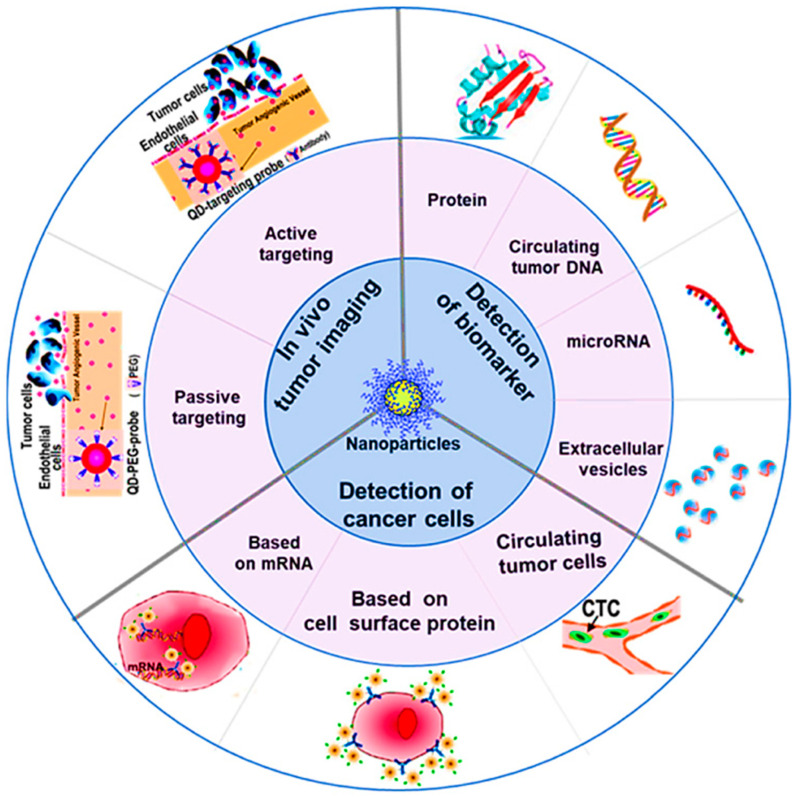
Mechanisms explored for the diagnosis of cancer using nanotechnology, reprinted from [86]. (Published under a Creative Commons Attribution 4.0 International license, https://creativecommons.org/licences/by/4.0, accessed on 1 May 2022).

Recently, biosensors have been widely explored in the recent era for diagnosing malignancies [84]. Biosensors involving the detection of various biomarkers have been regarded as a sensitive and selective tool for the diagnosis of cancer; nevertheless, the technique has been regarded as unsuitable for early diagnosis because of the decreased level of biomarkers in blood or tissue at the very initial stages of the disease [84,85]. The work of Zhang and co-workers presented several methods that have directly been used in BC diagnosis (Figure 1) [86].

### 2.1. Biomarkers for BC Detection

Various biomarkers have been exploited during the initial diagnosis of BC [87]. The method of diagnosis becomes much more valuable depending on how earlier and accurately the biomarker can be detected. Various tumor antigens have been investigated that may elicit a response specific to that tumor and, therefore, can be employed in the initial recognition of cancer. Different approaches were studied to identify tumor antigens [88]. Scientists have suggested a proteomics-based approach for detecting autoantibodies to proteins isolated from tumor lysate that may help identify antigenicity related to post-translational modification of tumor cell proteins. Several autoantibodies have been reported to be biomarkers for BC. RS/DJ-1 has been identified in 13.3% of newly recognized BC patients [89]. In addition, 15% of patients with a poor prognosis for BC had elevated levels of p53 autoantibodies; however, the biomarker was seen in malignancies other than BC [90]. Thus, p53 does not offer BC-specific autoimmunity [91]. Specific heat shock proteins (HSP) such as HSP 60 and HSP90 have also been recognized to elicit the BC-specific immune response [92]. Another serum protein, CA 15-3, was identified to be a circulating biomarker for BC [93]. Researchers have used this protein to detect BC recurrences and monitor treatment for metastatic cancer. The CA 15-3 concentration also had a prognostic impact at the initial stage [94].

HER/neu, a human epidermal growth factor receptor 2, has been recognized as a critical determinant in malignant alterations in BC that can indicate its destitute prognosis [95]. Specific ductal proteins have been isolated from nipple exudate and were identified as important BC biomarkers. Among various ductal proteins, lipophilin-B, beta-globin, hemopexin, and vitamin-D-binding protein have been found overexpressed in nipple exudate from samples of tumor-bearing breasts [96]. Several studies have also described the use of abnormally expressed miRNA-21 for early recognition of BC. NP or enzyme-labeled miRNAs have been found to have high sensitivity via miRNA hybridization [87,97].

### 2.2. Nanotechnology for Early Detection of BC

There has been extensive research on using nanotechnology for early cancer detection [98,99].

#### 2.2.1. Graphene NPs

Due to its unique surface characteristics and simple surface functionalization, many applications of graphene and its derivatives have been introduced into the biomedical field [100]. Graphene-based nanomaterials possess inherent anticancer properties and can enhance cell adhesion and capture BC cells. Graphene may form toxic byproducts in tumor cells through oxidative stress and autophagy. Graphene nanomaterials have been shown to inhibit macrophage activity, causing oxidative damage [101]. Rostamabadi and coworkers have demonstrated an electrochemical method for detecting BC biomarker HER-2 utilizing a transformed carbon electrode. Graphene oxide and carbon nanotubes (single-walled) were densely packed with AuNPs and placed on the glassy carbon electrode. The electrode was functionalized by an HER-2-specific aptamer that selectively recognizes HER-2 at the electrode interface, leading to enhanced charge transfer resistance using ferro- or ferri-cyanide as an electrochemical probe. This method was found to be highly specific and reproducible in discriminating the serum of patients affected with BC from serum samples of healthy individuals [102]. Safavipour and colleagues have proposed MUC-1 apta-sensors based on TiO nanotubes coupled with graphene oxide for the electrochemical recognition of MFC-7 BC cells. The biosensor effectively detected MUC-1 biomarkers in clinical samples and, thus, can help detect BC cells [103].

Xu and coworkers have investigated nano-enzyme decorated graphene quantum dots for electrochemical recognition of H_2_O_2_ in clinical BC analysis. The nanocarrier system exhibited high sensitivity and good biocompatibility for instantaneous tracing of H_2_O_2_ released from varying BC cells [104]. Graphene-based nanomaterials show high drug-loading capacity, easy functionalization, high target selectivity, and chemo sensitization. Graphene-based nanomaterials can be used in BC diagnosis and treatment due to their distinctive structures and attractive physicochemical properties.

#### 2.2.2. Mesoporous Silica NPs

In addition to chemotherapy, photodynamic therapy (PDT) and photothermal therapy (PTT) can be used as a non-invasive therapeutic strategy as a complement to overcome the deficiency of monotherapy. As a result of light exposure, photoactive therapeutics (photosensitizers) can produce reactive oxygen species (ROS) or hyperthermia, thus killing cancer cells. Mesoporous silica NPs are suitable for multi-drug loading since they have a large surface area, pore size, and volume, making them a suitable target for photo-chemotherapy. The structural merits of MSNs have generated considerable attention as potential partners for PDT in recent years. In solid tumors, many photosensitizers aggregate easily, reducing their efficacy, and have poor intracellular uptake, making them unsuitable for use. The incorporation of MSNs prevents PSs from aggregating and improves their targeting ability and biocompatibility, resulting in fewer side effects and stronger anti-cancer effects.

Wang and coworkers have proposed mucin-1 protein (MUC-1) targeted magnetic silica-based mesoporous NPs to capture MFC-7 cells. These silica NPs were coupled with a folate-receptor-directed fluorescent probe by conjugating folic acid and fluorescein isothiocyanate over the surface of bovine serum albumin for selective and specific labeling of folate receptors in HER-2over-expressed MCF-7 cells. The quantitative assay developed showed superior specificity and sensitivity towards MCF-7 cells [105]. Chen and colleagues have developed anti-HER-2 single-chain variable segment functionalized ultra-small silica nanostructures to increase tumor-specific targeting efficiency and improve renal clearance. This nanostructure was proposed to be highly efficient in imaging BC and an ideal candidate for delivering therapeutic agents to the desired region [106]. 

Qiao and coworkers have explored pH-responsive plumbagin-loaded theranostic NPs composed of mesoporous silica-covered gadolinium-III, conjugated with zoledronic acid for early recognition of BC-associated bone metastasis with increased sensitivity and selectivity [107].

#### 2.2.3. AuNPs

Salahandish and coworkers have demonstrated gold and silver NPs grafted graphene and nanostructured polyaniline for highly selective biosensing and a wide linear response range. The nanostructures were found to be ultrasensitive for label-free sensing of BC cells. These nano-biosensors had a faster response rate and a detection limit as low as 2-cells/mL for the SKBR-3 cell line. The sensor also exhibited a high detection efficiency of 90% [108].

In another experiment by Saeed et al., developed an electrochemical DNA biosensor based on AuNPs-modified graphene oxide for simultaneous detection detect CD24, a new prognostic marker in BC, and a transmembrane protein tyrosine kinase (ERBB2). The developed DNA nanosensor utilized a sandwich-type detection method, and amperometric detection was used to assess the sensor’s response. For this purpose, the gold nanoparticles were grafted onto the glassy carbon after being attached to graphene oxide. Surface immobilization was used to attach thiolated nucleic acid capture probes. Because of the amplification of the signal that was obtained, sensitive detection of both BC markers (ERBB2c and CD24c) was possible. The detection limit was found to be 0.16 nM and 0.23 nM for ERBB2 and CD24, respectively [109]. We believe that it would be a very encouraging approach to detect more BC markers at the same time.

Dong and coworkers have investigated HER-2 functionalized magnetic gold-shelled poly (lactic-co-glycolic acid) nanocarriers for ultrasound or magnetic resonance imaging and BC photothermal therapy. The nanocarrier system exhibited a receptor-specific binding HER-2 overexpressed in BC cells (SKBR-3) with a significantly high binding affinity. In vitro studies revealed that NPs possessed both enhanced ultrasound and magnetic resonance imaging properties [110]. Photothermal cytotoxicity experiments revealed multifunctional nanocarriers to have excellent photo-absorption and thus can be beneficial in the photothermal therapy of BC [110]. Rao and coworkers have suggested gold nanocluster-loaded functionalized liposomes for early recognition of HER-2 overexpressed BC cells [111]. In another study, an aptamer-based bipolar electrode system modified by AuNPs was used to amplify the signals, and the sensor can detect overexpressed nucleolin in BC (MFC-7) cells. The apta-sensors were found to have low cost, high sensitivity, and selectivity towards BC cells [112]. AuNPs functionalized with copper frameworks were also investigated for BC sensing with quaternary chalcogenide-platinum-doped graphitic carbon nitride (g-C_3_N_4_). Cu_2_ZnSnS_4_ NPs can be used for the detection of HER-2 in BC [113].

**Figure 2:**
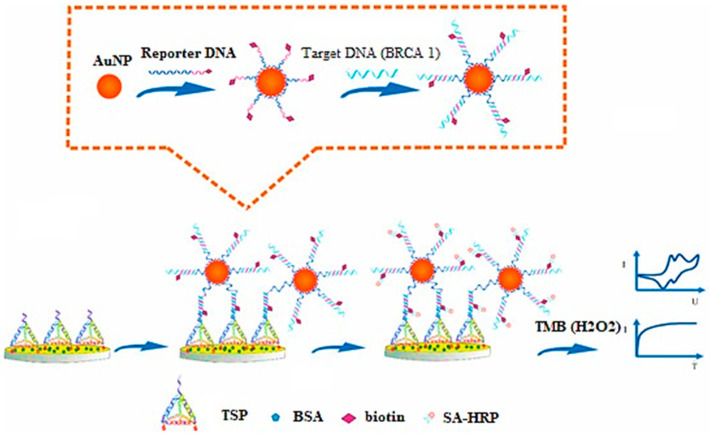
Principle for development of DNA-based electrochemical gold nano-sensors, reprinted from [115].

Tian and associates have investigated AuNPs for electrochemical sensing (label-free) of microRNA-21 (miRNA-21) via a redox indicator. These sensors exhibited high sensitivity, selectivity, and reproducibility in blood serum samples [114]. Feng and coworkers have developed an electrochemical AuNP-based biosensor for sensitive and selective detection of BARC-1 to form a sandwich-type assembly (Figure 2) [115].

#### 2.2.4. Silver NPs

Arteaga and coworkers have utilized silver NPs (citrate reduced) as a substrate for surface-enhanced Raman spectroscopy to quantify BC-associated elevation in sialic acid in saliva. This simplified test exhibited a 94% sensitivity and 98% specificity for the diagnosis of the BC patient, with a cut-off concentration of sialic acid assumed to be >7 mg/dL [116].

In another study, label-free geno-sensors were developed for the detection of BC-specific biomarker miRNA-21. The sensor was based on graphene functionalized silver NPs, resulting in significant electrochemical signal amplification. This biosensor was found to be very reproducible in assessing blood samples and can be used for direct recognition of miRNA-21 during the early stages of BC without the need for any sample preparation or RNA extraction [117].

#### 2.2.5. Iron-Oxide NPs

Albernaz and coworkers have investigated nano-radio-labeled superparamagnetic iron oxide NPs. These NPs were further conjugated with diethylene triamine penta-acetic acid labeled with technetium 99 and gallium 68 for SPECT and PET. The results demonstrated a high accumulation of nanocarrier systems in tumorous cells compared to normal cells, giving a clear contrast image by both techniques (SPECT and PET) [118].

Hsu and coworkers have proposed a multimodal imaging probe, called “all in one NP (AION)”, which is developed by combining near-infra-red fluorophore, silver sulfide NPs, and iron oxide NPs into PEGylated micelles. The AION exhibited a minimal release of silver ions leading to a significant decrease in associate cytotoxicity. A strong contrast was generated compared to all imaging modalities and exhibited a strong contrast when injected intravenously for in vivo tumor imaging. Thus, AION was suggested as an excellent candidate for BC detection with various imaging opportunities [119].

In 2021, Li and colleagues developed a motif (RXDLXXL)-linked arginine-glycine-aspartate nano-peptide and conjugated it with superparamagnetic iron oxide NPs (cFK-9-USPIO) for molecular imaging of integrin protein (αvβ6), overexpressed in BC. In comparison to the controls, the in vivo MRI of four nude mice carrying T1 xenograft tumors revealed a significant decline in T2 signal intensity in the BC tissue. Prussian blue staining provided additional confirmation that v6 integrin-targeted NPs had specifically accumulated in 4T1 BC cells. Comparatively, significantly fewer particles were seen in the 4 T1 tumors of mice that had been injected with control USPIO [120]. We believe that these findings provided solid evidence for the usage of integrin v6-targeted NPs to be applied in magnetic resonance molecular imaging and help target v6-overexpressed BC cells.

In another study, ultra-small iron-oxide NPs coupled with BRBP-1 were investigated for NIRF and MRI of BC-associated brain metastasis. The peptide-modified nanocarrier system was found to enhance the imaging signal induced by targeting the ability of BRBP-1, thereby increasing the potential of nanocarriers for the diagnosis of brain metastasis associated with BC [121].

Semkina and coworkers have studied anti-vascular endothelial growth factor coupled iron-oxide NPs intended for targeted transport of DOX in the murine breast adenocarcinoma 4T1 cell line. After 24 h, MRI was used to study how NPs accumulated in cancer cells, demonstrating that the carrier system can simultaneously deliver drugs and diagnose cancer [122]. We believe DOX is easily delivered to tumor cells due to the conjugation of anti-vascular endothelial growth factor (VEGF) antibodies with bovine serum albumin-coated PEGylated magnetic NPs. This excellent strategy can be pursued in future BC detection approaches.

Pacheco and coworkers have proposed molecularly imprinted polymeric sensors to sense and quantify HER-ECD. The quantification and detection limits were 1.6 ng/L and 5.2 ng/mL, respectively. The biomarker was found to be more selective in comparison to other protein biomarkers [123]. Zhang and coworkers have improved the F19 MRI sensitivity by developing peptide aptamer conjugated hyper-branched perfluoropolyether NPs for BC-specific detection. The NPs enhanced the mobility of fluorinated segments, thus increasing the sensitivity of MRI imaging. The conjugation of protein aptamer exhibited improved tumor targeting efficiency of NPs along with improved tumor penetration [124]. Wojtynek and coworkers have demonstrated self-assembled hyaluronic acid (HA) NPs encapsulating indocyanine green for BC imaging to enhance intraoperative contrast. The NPs enhanced the intraoperative contrast and helped identify small and occult lesions and, hence, can significantly improve surgical outcomes relevant to BC [125].

Jin and coworkers have developed a peptide (cyclic arginine-glycine-aspartic acid) conjugated polymeric NPs using poly(2-methoxy-5-(2-ethyl-hexyloxy)-1,4-phenylene vinylene) as a photosensitizer targeting TNBCs. The polymeric nanoconjugates exhibited bright flourescence, increased stability, and could generate reactive oxygen species on light irradiation. It was demonstrated that such nanoparticulate systems are useful as diagnostic tools for clinical use [126].

#### 2.2.6. Miscellaneous Nanocarriers

Zhang and coworkers have explored tumor-derived exosomes as biomarkers for detecting MCF-7 cells by targeting overexpressed mucin-1. The proposed on–off apta-sensors were turned on in the presence of mucin-1 on tumor-derived, resulting in fluorescence signal emission. The exosome-based biosensor successfully quantified a small number of samples, making them highly sensitive sensors [127]. Xu and associates developed allochroic acid NPs to detect overexpressed ERs, progesterone receptors, and HER-2 in BC using pH indicators. Further functionalization with bovine serum albumin and antibody was carried out to increase the dispersity of NPs. Moreover, the assay was coupled with a smartphone to ensure point-of-care analysis [128].

Mohammadniaei and colleagues have developed a new electrochemical biosensor using a topological illustrator and metallic DNA. Bismuth selenide NPs were prepared and sandwiched between an Au electrode, and Au deposited a thin layer of bismuth selenide. This was followed by immobilizing eight silver ion-mediated dsDNA onto the substrate to detect H_2_O_2_ liberated from BC cells. The bismuth selenide NPs were regarded as electrochemical signal boosters, while the Au coating added to the stability of these signals. The proposed biosensors offered a low detection limit (10 × 10^−9^ M) along with the excellent capacity to distinguish two variations of BC cells (MFC-7 and MDA-MB-231) contingent on the variation in H_2_O_2_ generation [129].

## 3. Multi-Functionalized Nanocarriers for BC Therapy

**Figure 3:**
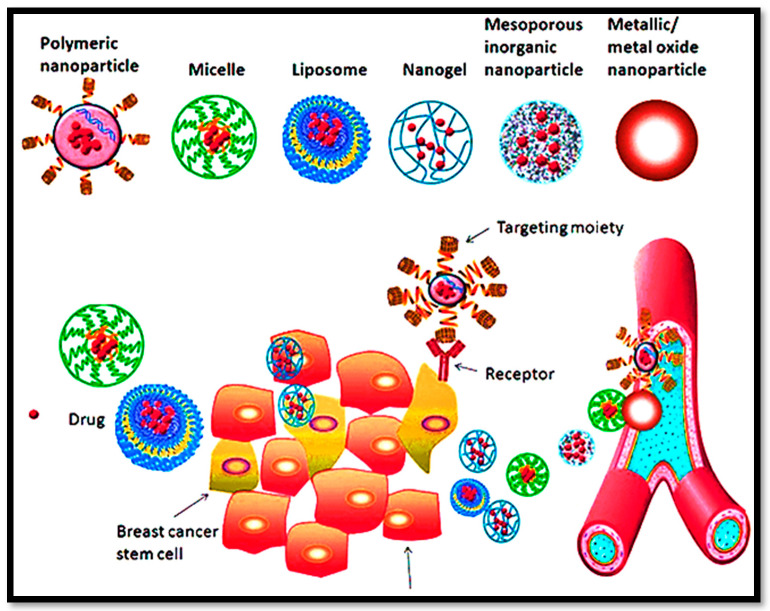
The proficiency of multifunctional nanocargoes for their versatility and expected clinical impact in BC management.

Multifunctional nanocarriers include polymeric NPs, self-nanoemulsifying drug delivery systems (SNEDDS), liposomes, and mesoporous inorganic NPs. The nanocarriers are supposed to permeate through the intestinal mucosa [130,131,132,133,134,135,136]. These systems improve oral drug absorption via paracellular transport, P-glycoprotein efflux inhibition, mucoadhesion, receptor-mediated endocytosis, and pinocytosis. In addition, the targeting approach based on lymphatic uptake is also interesting, as shown in Figure 3. A ligand-modified surface on nanocarriers contributes significantly to their stability and their ability to deliver targeted drugs [137,138].

**Figure 4:**
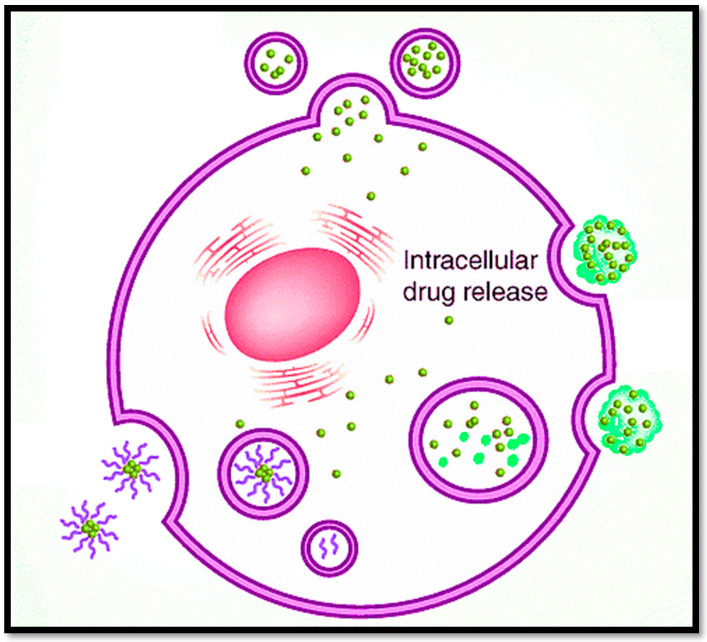
Multi-functionalized nanocarriers release the drug in a specific environment inside the tumor for targeted intracellular drug release.

The multi-functional nanocarriers can release the drug in a specific redox-triggered environment for intra-tumoral drug release, as shown in Figure 4, to maintain the target plasma concentration [139]. A targeted role for nanocarriers in BCs is provided in Table 1, and recent methods for nanostructures with specific relevance to BC are summarized in Table 2.

### 3.1. Ligand-Based Core–Shell NPs

Cancers of the breast are the world’s deadliest diseases, with a higher death rate among women. BC treatment is often associated with using oral chemotherapeutics in terms of radiation therapy, chemotherapy, hormonal therapy, and multi-functionalized ligand-mediated therapy [151]. Additionally, physical barriers to drug diffusion and insufficient delivery of drug concentration to the tumor are also major challenges [152]. The heterogeneity of tumor mass allows these approaches to transform, and it is necessary to target tumors more selectively [153]. In this connection, Dávid Kovács and co-workers also described the fact in detail that metallic-based core–shell NPs endorsed the efficacy in treatment against breast tumor metastasis. Therefore, they developed hybrid metallic NPs by adjunction of gold and silver NPs in the form of core–shell nanostructures. Hybrid NPs were synthesized by chemical reduction via using sodium borohydride. The results showed that targeted delivery of hybrid metallic core–shell NPs in the TME leads to the weakening of progressive tumor behavior of cancer-associated fibroblasts (CAFs) [147]. Darfarin and co-workers developed gold-silicon oxide shell-core NPs by conjugating gold NPs with silica NPs via amination and thiol-functionalization. The study concluded that gold-silicon core–shell NPs impart great efficacy towards MCF-7 BC cells via mega-voltage irradiation.

For the development of NPs, Zhang and associates [140] used electrostatic deposition and antisolvent precipitation. For the purification of Honokiol-free samples, centrifugation was performed. The synthesized nanostructures were analyzed via TEM, particle size determination, PDI and encapsulation efficiency, dissolution assay, as well as advanced cellular studies, in vitro Western blotting analysis, inhibitory effect, in vivo tissue distribution of NPs by living to image, in vivo therapeutic efficacy and toxicity studies. The results concluded that manufactured NPs resulted in 210 nm size, having a negative charge with improved anti-proliferative and pro-apoptotic changes against 4T1 cells. The mechanistic approach of HA-Zein-HNK was downregulation of the Vimentin expressions and upregulation of the E-cadherin expressions. In conclusion, it can be believed that HA-Zein NPs can serve as a promising approach in HNK delivery for metastatic BC therapy [140].

### 3.2. Dual pH-Responsive Polymeric NPs

L. Palanikumar and the research group developed biodegradable pH-responsive NPs conjugated with a polymeric system comprising poly(lactic-co-glycolic acid (PLGA). This polymeric system was then coated with bovine serum albumin (BSA). The important feature of this formulation was the inhibition of the macrophages, which causes the inhibition of recognition targets. Moreover, after the uptake of NPs, intracellular microenvironment conditions lead to degradation of NPs and proficient anti-cancer activity against BC cell lines [148].

Similarly, Zhihao Guo and co-workers developed a tailor-made 2,3-dimethyl maleic-anhydride-poly(ethylene glycol)-*ε*-poly-l-lysine-DOX/lapatinib polymeric nanoplatform for encapsulation of anti-cancer drug lapatinib for its conversion into switchable charge based dual pH-responsive NPs. Advanced physicochemical properties of novel NP conjugates lead to stability in the circulation of physiological conditions. However, charge switching capability from negative to positive charge leads to high-level sensitivity in the slightly acidic TME, facilitating strong muco-penetration [149].

Liu and coworkers [141] developed dual pH-responsive multi-functionalized nanocarriers via combining immunotherapy and chemotherapy based on poly(L-histidine) and HA for co-loading R848 (immune-regulator) and DOX via different encapsulation modes. Therefore, HA was initially reacted with succinic dihydrazide (SDH) by adding EDC and NHS as reaction activators to synthesize HA-SDH conjugate. The conjugate mixture was then dialyzed against deionized water, followed by lyophilization. To obtain a red cotton wool-like product, DOX was further conjugated with HA-SDH via an acid-cleavable hydrazine bond. Furthermore, PHIS and R848 nano-cores were formed by the nanoprecipitation method to obtain a slightly milky solution. The unloaded R848 was removed via ultrafiltration. The synthesized nano-cores were analyzed and characterized via infrared (IR) spectroscopy, proton nuclear magnetic resonance (1H NMR), drug loading, dissolution mechanistic, evaluation of maturation of DC2.4 cells, assessment of activation of advanced BC cell lines by flow cytometry, cellular uptakes, and intracellular locations, cell viability assay, in vivo pharmacokinetics, as well as biodistribution studies. Results concluded that the above-discussed combination is a promising novel therapy for cancer.

Moreover, the ionization of PHIS in the TME converts its hydrophobicity into hydrophilicity, which is very helpful in exerting immune regulation. Hydrazone bond breakage at endosomal pH accelerated the release of DOX, exerting cytotoxic effects. HA-DOX was overexpressed in BC cells and resulted in the internalization and inhibition of cell growth. In summary, this multi-functionalized nano-core system could deliver precisely in the TME and BC cells to achieve synergistic effects for excellent tumor-targeting ability against BC [141].

### 3.3. Mesoporous Carbon NPs

Carbon-based NPs can be synthesized via different approaches based on their application [154]. Therefore, Fan and coworkers [142] developed Resveratrol (RES)-loaded mesoporous carbon NPs as a promising approach against metastatic BC. However, successfully synthesized mesoporous oxidized carbon NPs (oMCNs) using mild oxidation techniques enabled the RES to be loaded with high efficiency. Synthesized RES-oMCNs polymer composites were characterized based on particle size determination, poly dispersity index, zeta potential, FTIR, XRD, SEM, and TEM. Then, the formulation was characterized in terms of some parameters, including dissolution, solubility studies, cell culture, cellular uptake, in vitro cytotoxicity assay, as well as cell apoptosis studies, including flow cytometry analysis and Western blot assay. This study found that oMCNs had a size below 200 nm, excellent water dispersion, and good encapsulation. OMCNs exhibited biocompatibility and excellent cellular uptake because of the preferential uptake of NPs owing to increased solubility of oMCNs-RES compared to the pure RES. Sustained drug release referred to the drug release only at lysosomal pH to improve the targeting capability of the therapeutic moiety. Moreover, in vitro cytotoxicity and apoptosis analysis showed caspase-3 protein cleavage in TNBC cell lines, respectively, thus showing greater interest in inducing anti-cancer activity against various metastatic cancer cell lines [142].

Additionally, Abid Hussain and Shengrong Guo investigated a new controlled system to release drugs from mesoporous carbon NPs (MCNs). The surface of the NPs was decorated with natural sophorolipids (SLPD). The pores were developed on the MSNs for inducing photothermal activity and encapsulating a chemotherapeutic drug (DOX). This advanced nanoparticulate system acts as a checkpoint for trapping DOX inside the pores of mesoporous carbon NPs and triggering its release in the TME via NIR irradiation. As a result, they demonstrated that this system is highly effective against BC cell lines [155].

### 3.4. Self-Nano Emulsifying Drug Delivery System (SNEDDS)

In 2019, Batool and collaborators synthesized SNEDDS of tamoxifen (Tmx) for targeting BC [143]. Following the previous method, thiolated hyaluronic acid (THA) was prepared with a few modifications [156]. Furthermore, papain (Pap), HA, and lithocholic acid (LCA) conjugate was prepared via chemically grafting lithocholic acid on papain-modified THA via the amide bond formation [157]. However, the novel polymer was initially characterized based on swelling, disulfide bond formation, and conjugation. However, this polymer conjugate was further linked with Tmxto form Tmx-PAP-HA-ss-LCA SNEDDS. Other characterization techniques included FTIR, XRD, DSC, TGA, solubility studies, preliminary screening of surfactants, preliminary screening of co-surfactants, construction of pseudo ternary phase diagram, percentage transmittance, dispersibility test, saturation solubility, robustness to dilution, cloud point measurement, physicochemical tests, and drug content determination. However, ex vivo characterization includes mucoadhesion, permeation study and P-gp efflux pump analysis, biocompatibility studies, and histological analysis. In addition, stability was measured over a six-month period in order to evaluate any discrepancies. Furthermore, SNEDDS showed stabilized encapsulation of the polymer. In vitro dissolution directed around 85% drug release within 48 h. The permeation study showed enhanced permeation of SNEDDS compared to standard drugs. Therefore, SNEDDS was muco-penetrating and showed anti-proliferative activity against BC cell lines [143].

Moreover, we believe that solid SNEDDS can improve patient compliance and tackle the limitations associated with liquid SNEDDS capsules. However, the selection of SNEDDS components is heavily influenced by their physicochemical features, drug solubility, and physiological fate.

### 3.5. Biodegradable Boron Nitride NPs

In 2019, Le and coworkers developed the advanced and on-demand technique of biodegradable boron nitride NPs (BNNPs) for specified targeting against negative BC cells via neutron capture therapy of boron [144]. For the NPs synthesis, researchers grounded melamine and boric acid (H_3_BO_3_) at a molar ratio of 1:6 to convert them into a powdered form. These precursors were then heated under airflow in the horizontal furnace. The resulting crude samples were then centrifuged to remove impurities, followed by scattering in the presence of 25 mL of H_2_O_2_ and then transferred to a Teflon-lined autoclave for thermal treatment at 120 °C for 24 h. Furthermore, the dispersions were cooled at room temperature and subsequently centrifuged at 4000 rpm for 10 min, followed by vacuum drying overnight, resulting in the synthesis of BNNPs. These NPs have been characterized via vitamin C triggered degradation, cellular uptake, PET imaging, biodistribution in tumor-bearing mice, degradation behavior in vitro, degradation behavior in vivo, tumor-bearing animal models, radiolabeling, in vivo Boron Neutron Capture Therapy (BNCT), and histological studies. Results concluded that in PET imaging, the coated BNNPs exhibited high tumor boron accumulation while maintaining an excellent tumor to non-tumor ratio, and BNNPs were found to be rapidly cleared from major organs according to ex vivo ICP-OES analysis. Furthermore, when compared with the control group, animals treated with BNNPs showed tumor growth suppression with negligible side effects. This novel strategy not only employed the high boron content of BNNPs but also efficaciously completed an on-demand degradation of BNNPs to avoid the potential toxicity caused by the long-term accumulation of NPs [144].

### 3.6. Polymeric Nanogels

Overexpression of estrogen hormone is linked to BC, and anti-estrogen therapies involving tamoxifen are the most commonly prescribed methods of treatment for it [158,159,160]. Nevertheless, breast tumors have been associated with resistance and non-targeted therapies, resulting in metastasis. However, using polyoxometalates (POMs) can provide a very effective treatment method for this condition [161]. POMs are discrete anionic metal-oxo clusters formed with early transition metals in the highest oxidation states, and over the past decades, POMs can be recognized for treating different types of cancers and infectious diseases [162]. In this regard, Pérez-Álvarez and coworkers [145] documented that low molecular weight chitosan was cross-linked in the inverse phase microemulsion medium forming nanogels. Characterization was performed via ^1^H-NMR and ^31^P-NMR spectroscopy, DLS, zeta potential, TEM, and atomic emission spectroscopy. Based on the findings of this research, synthesized nanocarriers are potentially useful in future treatments for BC [145]. Yi Zhang and co-workers developed dual-sensitive nanogels by inducing redox reactions. Dual sensitive nanogels were manufactured with the capability of rapidly dislodging the anti-cancer drug (DOX) for dual sensitization. These studies led to the development of drugs with extended-release times and rapid onset of action [163].

### 3.7. Ultrasound-Triggered Liposomes

The functionalization of liposomes with monoclonal antibodies is a potential strategy for increasing the targeting capability of the HER2, overexpressed in HER2-positive BC cells [164,165,166]. Therefore, calcein and DOX-loaded immuno-liposomes were functionalized by Elamir and co-workers with the monoclonal antibody trastuzumab (TRA) [146]. Liposomes were characterized for their size, phospholipid content and antibody conjugation, estimation of phospholipid content, and other advanced imaging techniques. It was concluded that combining immuno-liposomes and LFUS is a hopeful technique for targeted drug delivery in reducing the cytotoxicity of antineoplastic drugs [146].

We already know that anticancer medications can be delivered to the body via liposomes to either lessen the harmful effects of the drugs when administered on their own or lengthen the circulation time and effectiveness of the treatments [167]. According to the evidence given below, we think it is possible to direct aqueous contrast-enhancing chemicals that are loaded in liposomal carriers to the breast tissue and then use computed tomography to differentiate between normal and tumorous breast tissue. In this regard, Yonghong Song and co-workers developed emodin-based liposomal NPs. As it is reported in the literature that emodin possesses strong anti-cancer activity, but its efficacy is somehow compromised owing to the poor solubility and non-targeted delivery. Therefore, the specificity of the emodin was improved by incorporating it into the liposome’s lipid bilayer. Furthermore, the hydrophobic layer of the liposomes was concurrently loaded with high-performance ferromagnetic iron oxide nanocubes. Results concluded that magnetic field targeting leads to the efficacious and sensitive targeted killing of BC cell lines [150]. Yuko Okamoto et al. developed paclitaxel-based liposomes for the reason that paclitaxel is an insoluble anti-cancer drug and has issues of low solubilization and poor pharmacokinetics. Therefore, a liposomal formulation of paclitaxel within its aqueous core without adding organic solvents finally showed potent activity against BC cell lines [168]. Similarly, Snehal K. Shukla and her research team developed metformin hydrochloride (Met) encapsulated within liposomal vesicles via thin-film hydration technique by active and passive loading of drug-loaded lipid film. The metformin liposomal formulation leads to advanced therapeutic efficacy, i.e., an increased therapeutic outcome at the minimized dosage and profound apoptosis-induced activity in BC cells [169].

Various preclinical studies regarding NPs against BC involve methotrexate and beta carotene conjugated lipid polymer hybrid NPs for inducing BC by A Jain and co-workers [170]. Moreover, zein NPs coated with beta carotene can improve the anti-cancer activity and abolish the toxicity of methotrexate by the same group [171]. M. Khoobchandani’s research group utilized a new approach for targeting BC via green nanotechnology by conjugating NPs with the ayurvedic system for pilot human clinical investigations [172]. The advantages and disadvantages of different strategies against BC are shown in Table 3.

## 4. Theranostic Application of Nanocarriers in Management of BC

More accurate and targeted co-delivery of both therapeutic and diagnostic compounds is being pursued due to the last two decades’ worth of nanotechnological breakthroughs in biomedical research for cancer treatment using drug delivery carriers and contrast agents [173]. Nano-carriers are the key to achieving increased availability, tailored cellular absorption, and low toxicity. These unique nanocarriers are constructed to target the local BC cells while carrying all the required weapons (tracking probe, drugs, and ligand). A theranostic strategy that articulates the multifunctional molecules with a targeted delivery system for higher sensitivity, tracking, diagnostics, and therapy [174]. It is believed that tailoring nanostructures in a beneficial manner would tremendously help to simultaneously treat and diagnose BC in affected patients.

### 4.1. Inorganic NPs

A class of nanomaterials that are inert (resistant to corrosion/oxidation) and have distinct physical and sensing characteristics are inorganic NPs. For the identification of a variety of biomarkers, inorganic NPs, in particular silver and gold nanoparticles (AuNPs and AgNPs), are very precise and sensitive biosensors [175]. For example, an electrochemical nanosensor based on a three-screen-printed carbon electrode (3SPCE) array modified with graphene oxide, graphene quantum dots and gold nanoparticles (AuNPs/GQDs/GO) was successfully built to identify the breast cancer biomarkers miRNA-21, miRNA-155, and miRNA-210. Great performance for multiplexed miRNA sensing was disclosed by the developed AuNPs/GQDs/GO-based biosensor. With supersensitive low LODs of 0.04, 0.33, and 0.28 fM for the detection of miRNA-21, miRNA-155, and miRNA-210, respectively, it provided a broad linear dynamic range from 0.001 to 1000 pM. High applicability and selectivity were also shown for the identification of miRNAs in human serum samples. [176].

Proteins, nucleic acids, and other macromolecules are dephosphorylated by the enzyme alkaline phosphatase (ALP). It has the potential to be a biomarker for many different illnesses, including hepatobiliary, osteopenia, and breast cancer. In light of this, a colorimetric sensor for the ALP test was developed. This sensor is based on the enzyme’s ability to dephosphorylate the compound p-aminophenol phosphate (pAPP) into pAP. Silver nanoparticles (AgNPs) and Ag+ ions are present in a solution that has been made using a low concentration of NaBH4, and pAP mediates the growth of AgNPs by lowering the concentration of Ag+ ions to increase the intensity of localized surface plasmon resonance because pAPP is unable to cause a reduction of the remaining Ag+ because the hydroxyl is being covered by phosphate. The colorimetric detection of the pAP-mediated development of AgNPs in the presence of an ALP served as a proof of concept for the quantitative analysis of the ALP. AgNPs’ extremely sensitive enzymatic growth offered a lower limit of detection of 0.24 U/L and a larger dynamic linear range of 0.5–225 U/L than had previously been reported. For the ALP test in human blood, the application of pAP improved the sensor’s selectivity, resulting in a high recovery rate and high accuracy of 99.2 1.5 percent for the traditional addition technique [175].

### 4.2. Liposomes

**Figure 5:**
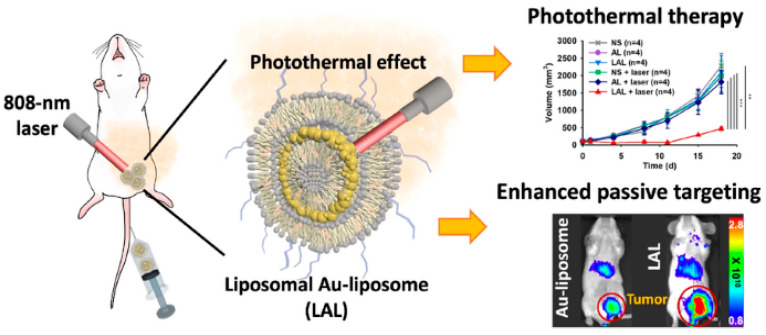
Theranostic dual-layered Au-liposome shown schematically for efficient BC targeting and photothermal treatment, reprinted from [177].

For instance, a liposomal layer was recently added to AL (LAL), followed by radiolabeling and the addition of polyethylene glycol (PEG), to create a theranostic dual-layered nanocomposite [177]. LAL’s in vivo stability is increased by functionalization with PEG, and in vivo imaging of LAL is made possible by radioisotope labeling. Functionalized LAL was stable under physiological circumstances, and in vivo positron emission tomography (PET) imaging of 64Cu-labeled LAL (64Cu-LAL) demonstrates an adequate blood circulation capacity and an efficient tumor-targeting potential of 16.4% ID g-1. Additionally, compared to intravenously injected AL, intravenously injected LAL demonstrated greater tumor targeting, in vivo temperature rise, and superior PTT action. LAL had a 3.9-fold better rate of tumor inhibition than AL [177]. Figure 5 shows a schematic of a theranostic dual-layered Au-liposome for photothermal treatment and efficient BC targeting.

In a related investigation, the effectiveness of hybrid liposomes (HL) as therapeutic agents and the capability of Indocyanine Green (ICG), a fluorescent probe containing HL, to identify malignancy in orthotopic graft model mice of BC (MDA-MB-453) were assessed. As a result of their therapeutic benefits and their capacity to identify (diagnose) cancer in an orthotopic graft model mice of BC, the results suggested that HL and HL/ICG might be theranostic targets [178].

### 4.3. Polymer NPs

Due to their excellent characteristics, conjugated polymer NPs (CPNs) have become a novel and promising group of BC theranostic weapons [179]. In this light, thio-phene-quinoxaline type conjugated polymers with three-four fluorine atoms on the repeat unit were used to manufacture nanoprecipitated and encapsulated aqueous CPNs. The findings demonstrated that, in addition to the visible fluorescence on the triple-negative BC cells, only the nanoprecipitated CPNs with the three fluorine atoms displayed excellent intracellular uptake to all of the epithelial cell types evaluated. Additionally, an examination of the CPNs’ effects on cell proliferation and death of all cell lines with the matching antibiotic staurosporine was conducted, suggesting a possible therapeutic effect of the NPs under investigation [179].

**Figure 6:**
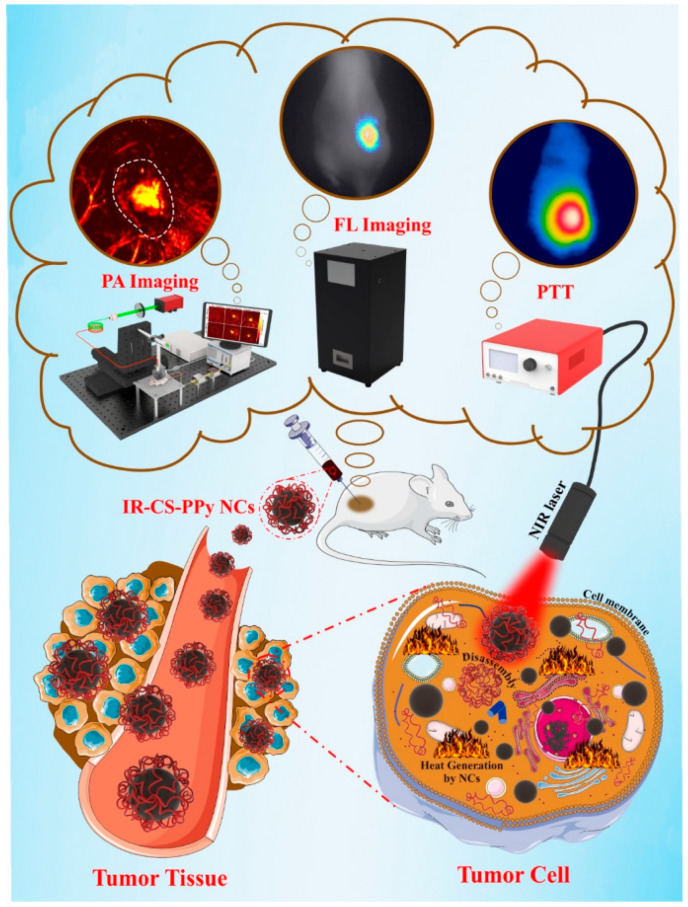
Dual-channel photoacoustic/fluorescence imaging-guided photothermal treatment using IR-CS-PPy NCs, reprinted from [180].

A multimodal polymeric contrast agent and imaging techniques have the potential to make important advances in the area of biomedicine [180]. To address this, in order to produce a theragnostic agent for dual-modal imaging using both fluorescence (FL) and photoacoustic (PA) imaging, IR783 attached chitosan-polypyrrole nanocomposites (IR-CS-PPy NCs) were created. The in vitro photothermal findings show that IR-CS-PPy NCs may effectively destroy MDA-MB-231 BC cells when exposed to an 808 nm NIR laser. The in vivo PTT investigation showed that IR-CS-PPy NCs completely destroyed the tumor tissues, preventing any further regrowth [180]. Figure 6 shows a schematic of IR-CS-PPy NCs used in dual-channel photoacoustic/fluorescence imaging-guided photothermal treatment.

We believe that patients diagnosed with BC benefit from an improved prognostic evaluation when multimodal imaging and therapy features are combined. The above dual systems worked fine and can be applied for BC imaging and treatment.

## 5. Conclusions and Future Perspectives

Advancements in nanomedicine have brought nanotechnology-based methods to the forefront of cancer research. Multi-functionalized nanostructures with intelligent design and unique physicochemical properties have revolutionized targeted drug delivery and shown great potential in BC theranostics. An overview of BC nanomedicine’s experimental and clinical accomplishments using active targeting and flexible biomedical nanostructures is presented in the paper. To give an example (graphene, silver, gold, silica, and oxide NPs) and their potential applications in BC imaging, drug delivery, combination therapy, and theranostics. Furthermore, it can be concluded that nanovesicles such as liposomes, nanogels, polymeric nanocarriers, mesoporous inorganics, biodegradable boron nitride, self-nanoemulsions, core–shell NPs, etc. have set a benchmark in the prevention of multiple BC subtypes by targeting drug delivery. Even though preclinical results indicate that these nanostructures are clinically relevant, several problems still exist and need to be tackled before their clinical application can be sought. 

Moreover, the surface properties of a nanosystem determine its behavior and interaction with proteins and cells [181]. Several surface properties (a charge, hydrophobicity, functional groups, etc.) contribute greatly to NP’s stability and opsonization. It has been demonstrated that poly(ethylene glycol) (PEG) and other polymers can serve as hydrophilic surfaces and protect NPs from opsonization and immune recognition [182]. The PEGylated Liposomal DOX (Doxil^®^) exhibits a prolonged half-life, increased tumor drug concentration, and superior antitumor efficacy compared to conventional DOX [183]. Targeting BC-associated fibroblasts which control the surrounding TME, the use of nanocages comprised of different compounds (sulfur or nitrogen-doped carbon, silicon, ferritin, gold, zinc, copper, metal–organic frameworks, PtCu3, etc.) is also a promising nanotherapeutic strategy for the combined chemotherapy and photodynamic therapy of BC. Furthermore, to fully characterize nanosystems, higher-level tests should be required compared to standard pharmaceuticals due to their complex nature. Another strategy can be taking advantage of biomimetic NPs to fight BC. The intracellular distribution of a variety of agents, including proteins and nucleic acid, can be facilitated by biomimetic NPs that feature diverse characteristics that are derived from the parent cell. As a result, biomimetic NPs have a significant potential to increase the therapeutic efficacy of the current nanodelivery systems, particularly concerning the development of individualized treatments for BC. Interestingly, the field of TME-related nano-delivery systems is rapidly evolving to overcome technical challenges and cost-ineffectiveness of using conventional BC therapies; hence, more efforts should be put into developing novel TME-targeted NPs in the feisty fight against this type of malignancy.

BC mortality rates can be significantly reduced by early detection of the disease with nanotechnology and therapeutics that are more effective. Nanomedicine promises several opportunities for both specific and multi-acting cancer treatments; nonetheless, the most important question is whether or not nanomedicine will be clinically transferrable in the near future. Clinical practice for BC has not been enhanced by nanostructures as expected. The main reason may be the absence of adequate preclinical models that effectively mimic, both spatially and physiologically, actual BC and its intricate interactions with the surrounding microenvironment. Because the vast majority of potentially effective drugs and therapeutic approaches developed in preclinical studies do not reach clinical trials, clinical translation cannot be carried out separately from this point. For clinical approval to happen more rapidly, a collaboration between academia and pharmaceutical companies will be beneficial to developing the field and improving clinical trials. Examining questions of long-term toxicity, biocompatibility, and atomic-level organization of these nanostructures is equally crucial before entering the market. Hopefully, cancer nanomedicine will enter its next phase of advancement by incorporating nanostructures into rationally designed therapies. As a result, new opportunities in BC nanomedicine may arise by developing innovative, high-level strategies and systems based on active targeting using a variety of nanostructures.

In our opinion, BC is mostly hormone-sensitive, employing both estrogen and progesterone sensitivity. Therefore, targeting the receptors of specific hormones through the coating of targeted ligands is the most effective method of halting BC tumor growth as an inducer for binding specifically to the receptor site. Additionally, shifting from traditional chemotherapy to a more effective targeted endocrine therapy can have a substantial impact. In this regard, we can safely assume that synergistic conjugation of chemotherapy and endocrine treatment will impart a significant effect and prove BC treatment as a breakthrough. Using nanoscale compositions of materials that have already been approved for use in humans is expected to be the first step in developing potential future nanomaterials in BC treatment. Before the significant potential of nanomedicine can be realized for the benefit of the affected patients, ongoing interdisciplinary collaborations between cancer biologists, toxicologists, bioengineers, material scientists, medical oncologists, and pharmacologists will be required.

## Figures and Tables

**Table 1:** Summary of multi-functionalized nanocarriers in treatment of BC.

Nanocarrier	Key Feature	Ref
Zein/HA core–shell NPs	HA-Zein NPs can serve as a promising approach in honokiol delivery for metastatic BC therapy	[140]
Dual pH-responsive multifunctional NPs	Dual pH-responsive multifunctional NPs for synergistic therapy against BC.	[141]
Resveratrol-loaded oxidized mesoporous carbon NPs	Resveratrol (RES) loaded MCNs can be an encouraging approach against metastatic TNBC.	[142]
HA-based SNEDDS	SNEDDS formulation was muco-penetrative as well as anti-proliferative towards BC cell lines.	[143]
Biodegradable Boron Nitride NPs	On-demand technique for NPs can be specified for targeting TNBCs through neutron capture therapy of boron	[144]
Chitosan nanogels	Chitosan nanogels were carriers for the polyoxometalates against metastatic BC	[145]
Ultrasound-triggered Herceptin liposomes	Favorable for targeted drug delivery in reducing the cytotoxicity of antineoplastic drugs.	[146]

**Table 2:** Methods for nanostructures in the form of a detailed table with specific interest to BC.

Nanoformulation	Methodology	References
Ligand-based core–shell NPs	Chemical reduction, amination, thiol functionalization, electrostatic deposition, and anti-solvent precipitation.	[147]
Dual pH-responsive polymeric NPs	Carbodiimide reactions	[148]
Mesoporous carbon NPs	Photothermal activity and mild oxidation	[142]
HA based SNEDDS	Chemical Reduction, Carbodiimide chemistry	[143]
Biodegradable Boron Nitride NPs	Neutron capture therapy of boron	[144]
Polymeric Nanogels	inverse phase microemulsion medium, redox-reaction	[149]
Liposomes	Thin film hydration	[150]

**Table 3:** Advantages and disadvantages of different strategies against BC.

Nanocarrier	Advantages	Disadvantages
Ligand-based core–shell NPs	A promising approach in honokiol delivery for metastatic BC therapy	Low mechanical resistance
Dual pH-responsive multifunctional NPs	Dual pH-responsive multifunctional NPs for synergistic therapy against BC.	Economical burden
Mesoporous carbon NPs	MCNs can be an encouraging approach against metastatic TNBC.	Anaphylactic reactions
HA-based SNEDDS	SNEDDS formulation was muco-penetrative as well as anti-proliferative towards BC cell lines.	Stability issues
Biodegradable Boron Nitride NPs	On-demand technique for NPs can be specified for targeting TNBCs through neutron capture therapy of boron	Non-broad-spectrum activity
Nanogels	Nanogels were carriers for the polyoxometalates against metastatic BC	Less encapsulation
Liposomes	Favorable for targeted drug delivery in reducing the cytotoxicity of antineoplastic drugs.	Costly, Difficult industrial scaling

## Data Availability

This article’s data sharing is not applicable as no new data were created or analyzed in this study.
